# An Overview of Fog Computing and Edge Computing Security and Privacy Issues

**DOI:** 10.3390/s21248226

**Published:** 2021-12-09

**Authors:** Ahmed M. Alwakeel

**Affiliations:** 1 Sensor Network and Cellular Systems Research Center, University of Tabuk, Tabuk 71491, Saudi Arabia; aalwakeel@ut.edu.sa; 2 Department of Information Technology, University of Tabuk, Tabuk 71491, Saudi Arabia

**Keywords:** cloud computing, fog computing, edge computing, cloud security, fog security, IoT, privacy of IoT

## Abstract

With the advancement of different technologies such as 5G networks and IoT the use of different cloud computing technologies became essential. Cloud computing allowed intensive data processing and warehousing solution. Two different new cloud technologies that inherit some of the traditional cloud computing paradigm are fog computing and edge computing that is aims to simplify some of the complexity of cloud computing and leverage the computing capabilities within the local network in order to preform computation tasks rather than carrying it to the cloud. This makes this technology fits with the properties of IoT systems. However, using such technology introduces several new security and privacy challenges that could be huge obstacle against implementing these technologies. In this paper, we survey some of the main security and privacy challenges that faces fog and edge computing illustrating how these security issues could affect the work and implementation of edge and fog computing. Moreover, we present several countermeasures to mitigate the effect of these security issues.

## 1. Introduction

Nowadays, cloud computing has become an effective solution to allow an on demand platform for processing and sorting a huge amount of data [1]. The implementation of cloud computing has spread across different areas including but not limited to education, finance manufacturing and healthcare [2,3]. As more systems start to rely on cloud computing, the need for new technologies that leverage the benefits of cloud computing but considered a lightweight solution without all the complexity of the cloud is needed to fit with the properties of lightweight systems such as IoT systems. Although devices in IoT systems can preform some indispensable tasks such as controlling, actuating and sensing these devices cannot accomplish complex and sophisticated tasks such as controlling large smart transportation systems and smart medical treatments. on the other hand. Many IoT applications are time critical applications which may require immediate decision to give the best possible performance an example of such applications are vehicular network and assisted healthcare application. As these applications cannot handle network latency, sending the data for processing to cloud resources may cause delay in decision making in such time critical application. Furthermore, Cloud computing is too complex for these devices to handle and it does not support some of the fundamental properties of IoT systems such as location awareness and bandwidth shortage. Two new technologies that can provide the benefits of cloud computing and yet addresses the special characteristics of IoT systems are Fog computing and Edge computing [4]. Although these new technologies open doors to enhancement and growth of IoT systems it introduced several security and privacy issues that may affect deployment and usage of IoT systems.

In this paper, we survey and list some of the main security and privacy concerns of the two technologies explaining how each attack may effect them as well as provide some countermeasures that could help mitigate or prevent these security concerns in order to achieve higher level of security and stable performance for the system. Although many attacks that affect fog and edge computing are inherited from cloud computing, many of the current solutions for cloud computing will not be effective or may not work properly as in normal cloud environment due to the unique properties of fog and edge computing. So we will highlight the uniqueness of these attacks in the realm of fog and edge computing in this paper as well.

Some authors in the literature have discussed the security concerns for edge computing as well as for fog computing. To our knowledge no other survey discuss the two technologies showing different attacks that could be related to both environment as well as which attacks may have higher impact on one specific environment. In [5], the authors did systematic analysis of some of the attacks that could affect fog computing showing the percentile of each attack happening in the environment. In [6] the author list some security concerns toward specific applications for fog computing. As for edge computing In [7], the authors discussed the security concerns in IoT applications that rely on edge computing. In [8], the authors survey security and privacy issues for edge computing and they classified the attacks based on the layer that the attack aims to effect. More surveys are listed regard edge and fog security in section four and five of this paper.

In order to have a better understanding of this topic and understand the differences between cloud computing, edge computing and Fog computing we will first provide an overview of cloud computing in general. Explaining different deployment methods for cloud computing fallowed by an overview of Fog computing and Edge computing showing how they differ from each other and traditional cloud. The following section will go over some of the security and privacy issues for the two technologies fallowed by countermeasure section where we will list some of the methods to mitigate these attacks for both fog and edge computing. Finally in the last section we will provide a conclusion and a future work.

### 1.1. A General Overview of Cloud Computing

Cloud computing allows high utilization of resources with high scalability and flexibility. With other advantages such as decreasing the power consumption and allow the entire services to be on demand so the users will have to pay only for the resources they will use as well as access from anywhere. There are different definition for cloud computing but we can consider the definition provided by NIST as one of the most broad definition which define cloud computing as “a model for enabling convenient, on demand network access to a shared of pool configurable computing resources (e.g., networks, servers, storage, applications, and services) that can be rapidly provisioned and released with minimal management effort or service provider interaction” cloud computing provide three different service models which are infrastructure as a service (IaaS), platform as a service (Paas) and software as a service (SaaS).

### 1.2. Cloud Computing Service Models

#### 1.2.1. Infrastructure as A Service (IaaS)

The Iaas model is the most abstract model for a cloud service provider simply provide servers and storage as a virtual entity through the cloud for their customers. In this service, models’ users can use the infrastructure to install their applications and operating system without having to worry about maintenance and operation of the underlying infrastructure. Moreover, users can enjoy scaling up or down their services without having to buy or update their actual hardware since cloud service is on demand service where users pay only for their usage. One of the most famous IaaS models is Amazon’s Ec2. Two example of use cases for IaaS model are date warehousing and big data analytics. IaaS provides the fallowing advantages:

- Considered to be the most flexible model from cloud computing.
- Cost effective in term of purchases of hardware based on consumption.
- The client has full control over the entire infrastructure.
- Highly customizable and scalable environment.

However, IaaS normally have higher security concerns over the other two models as the client is responsible of building the infrastructure which could create some threats the client isn’t aware of existence. Moreover, such models normally require additional training for the workforce in order to learn how to effectively control and manage the infrastructure including having to do additional tasks such as backup and update for the components of the infrastructure.

#### 1.2.2. Platform as A Service (PaaS)

This type of service model is all about providing an environment for users to deploy host, test and develop software application by consumers. In this type of models, a third party provider provide access to software and hardware tools to the user over the internet. PaaS allow freedom to the users from having to install hardware and software in order to develop their application moreover it could provide extra processing power that are hard to be acquired by single user instead users here pay on per use basis for the tools. PaaS provide the fallowing advantages:

- Cost effective and simple solution for development and deployment of apps.
- Unlimited scalability for the platform
- Automation of business policy
- Ease of migration toward hyper model.

However, some of the concerns related to PaaS is vendors lock-in where vendors may decide to make some changes to the requirements or of some solutions in the future without providing clear migration policies to the clients. Moreover, sometimes issues rise when users of this model try to integrate and connect data stored within off-premises cloud when using some components from legacy IT system.

#### 1.2.3. Software as A Service (SaaS)

SaaS is one of the most common service model of cloud computing that many users use on daily basis. This model is also known as cloud application services. In this model applications are delivered to the users over the internet without the need for user to install or update these applications. Moreover, most of the applications runs directly through the browser which add extra layer of flexibility and convenient to users of such model. SaaS provide the fallowing advantages:

- Cost effective solution to access to different applications without needing to update the hardware.
- Verity of solutions and services available to the users with one click.
- No need to upgrade the software as the user will always have access to the most recent version of the application.
- Ease of use.

**Figure 1:**
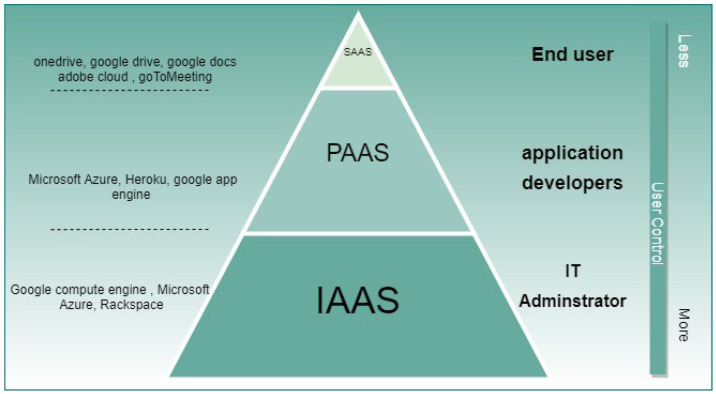
Cloud services level compression.

However, SaaS has created several concerns, such as lack of integration support for the application provided as a service with other applications that the users use locally. Additionally, normally SaaS provide minimal customization capabilities to the user since it’s normally provided as a one size fits all solution. Figure 1 shows the control level for users for each service level as well as some examples.

## 2. Fog Computing Overview

Unlike traditional cloud computing, fog computing—also known as fogging—focuses more on decentralization of the computer structure, located in between the devices that produces the actual data and the cloud. In fog computing the focus is on lowering the cloud computation capability to the edge of the network to be able to provide faster services to the users including communication service and software services. This comes handy in providing cloud solutions for high mobility technologies such as Internet of things (IoT) and vehicular ad hoc networks (VANET). Rather than having the devices connect through complex network infrastructure in fog computing the devices are normally connected directly to their destination. As a result, the connection will have much lower latency and better quality of service.

The concept of fog computing was proposed by Cisco Systems. Fog computing system was not introduced to replace cloud computing, rather it was introduced to fill the services gaps of cloud computing [9,10]. According to Cisco Systems, fog computing is a virtual platform that offers computation, storage and networking services to end devices from cloud computing data centers which are not specifically located at the network edge. Fog is different from cloud in a way that it has got end users closer to it as compared to cloud, to provide them services and respond to their demands in less amount of time. In cloud computing, computing, control and storage data are transported to the centralized cloud, whereas in fog computing, central and local computing, storage, and network management is balanced [11]. According to [12], fog computing is defined as a distributed computing technology where maximum operations are performed by virtualized and non-virtualized edge devices. It has some similar characteristics with cloud including non-latency aware processing and ability to store useful data for a longer time period by existing between the users and the cloud. The basic architecture of fog computing is similar to cloud computing, but its lower layers contain special components that are able to detect rare time response efficiently. Due to this feature, fog computing is used to control and improve health care department, traffic pattern, parking system and much more. Fog computing consists of disseminated framework consisting of a keen gadget that has certain application services outside the system. In other words, it gives authority to the gadgets to handle their associations and tasks any way they prefer best. Basically, fog computing is a focal layer residing between the cloud and the equipment, possessing enhanced information handling, investigation and capacity, which is achieved by reducing the amount of information to be transferred to the cloud [13]. Fog computing provides an improved administration and smooth client experience. It is basically a combination of hardware and software systems which has the power to monitor, control, and analyze data with extremely low latency [14]. In addition, Fog computing does not provide a permanent storage. It reduces the load on cloud by deleting unnecessary data into its computational storage, which also minimizes the cost. Fog devices are slightly different from the cloud devices in terms of their position and application for which they are installed.

Some of the differentiating factors include:

- If the deployment purpose of the device is customization of the end network services or sensing some data then the device is categorized as a fog device, otherwise it is a cloud device.
- If the device has limited computational capability and sensing ability, then it is classified as fog device.

Some of the features of fog computing include:

- Awareness regarding the location of edge.
- Very low latency.
- Mobility support.
- Real-time services.
- Good interactions.
- Heterogeneous nature.
- Inseparability.

**Figure 2:**
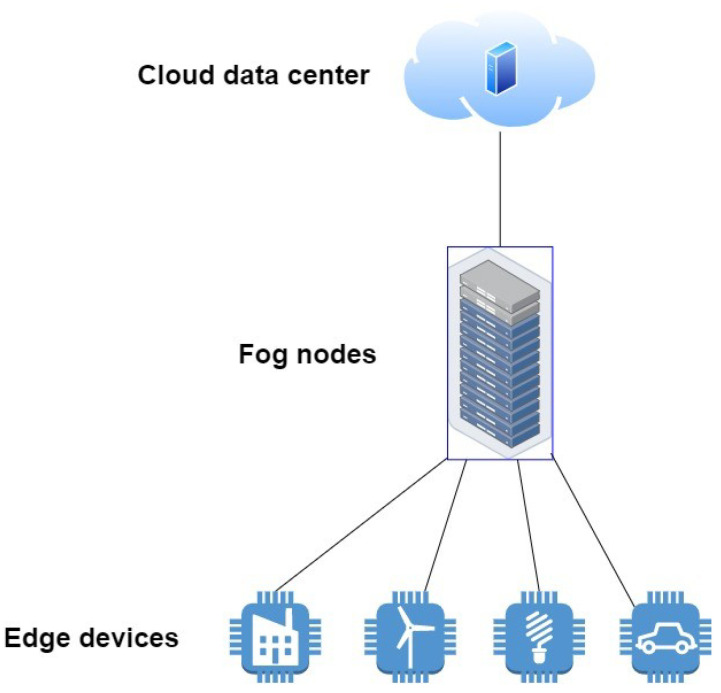
Cloud, fog and edge interconnection.

Most users now demand running applications that require heavy computational resources, which are beyond the processing capability of a mobile device, since it has very low processing speed. Besides low computational power, energy also becomes limited, since application processing are need to be offloaded to nearby cloud servers. Task offloading solves the issue of computational processing, but it is not feasible for time critical application. Hence, in such cases, the concept of fog computing is considered a good solution. However, fog computing also has some limitations such as low resources as compared to cloud, therefore, leading to high latency, energy consumption, load balancing, data management, and security threats. Figure 2 shows the three paradigms and how they interconnect.

## 3. Edge Computing Overview

In the past few years, there has been a change observed in the trend of computing which is pushing the service of clouds towards the edge of the networks. In other words, the computing operations and services are being shifted from network core to the network edge. This emerging technology is referred to as edge computing. Edge computing is different from fog computing technology with respect to the computing location. In fog computing network, local area network (LAN) performs the job of a gateway, whereas in an edge computing network, smart devices such as programmable automation controller (PACs) perform computing operations. The reason behind the evolution of edge computing concept is to overcome the high latency and energy consumption service issues of Cloud computing, and allow low-latency computation offloading services for resource-constrained devices and Internet-of-Things (IoT) applications. Moreover, it also provides the advantages of content caching and storage services which help in managing high network traffic.

The key feature of edge computing include:

- Very low latency.
- Reduced bandwidth limit.
- Flexibility in deployment.
- Automation.

Edge computing is the main domain which covers a family of related technologies as sub-domains such as cloudlet, mobile cloud computing, and multi-access edge computing. All these sub-domains are based on the concept that computational resources should be present at the edge where network encounters the real demand challenge, which leads to:

(1) Gain of fast access to the computation resources by the end users.
(2) Reducing the traffic load on network core significantly.

**Figure 3:**
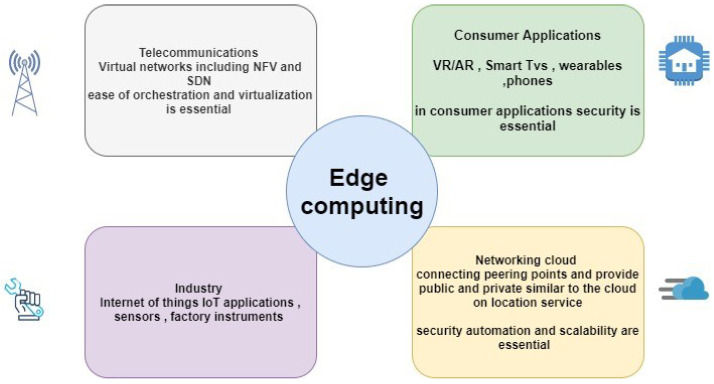
Edge computing applications.

Recent estimation shows that in the coming few years, there will be a deployment of tens of billions of Edge devices with exceptionally high processing speeds. This upcoming paradigm is called mobile edge computing (MEC), also referred to as multi-access edge computing (MEC) [13,15]. MEC is an emerging technology that provides storage and mobile computing resources at the edge of the network, for easy accessibility to the users and increase the efficiency of the edge computing [16]. It offers a low latency, high bandwidth, and real-time service environment that users can enjoy without any computational complexity [17]. In MEC architecture, the cloudlets holding data, also referred to as multitenant data centers, are placed near base stations (BSs) and access points (APs), so that users can easily run real-time applications without any delay and complex computing, on low-resource mobile devices with enhanced quality-of-service (QoS) [18]. Figure 3 shows some of the applications for edge computing.

While on the one hand, edge computing allows better experience and delay-free services, on the other hand, it also faces numerous challenges such as delay, bandwidth cost, high energy consumption, computational offloading, security, and quality of service (QoS). Edge computing can provide many solutions for different fields including factory floors, telecommunication companies and industrial automation.

Now that we have a good understanding of the concept of cloud computing, edge computing and fog computing and the difference between each of them in the next chapter we will discuss the security of both fog computing and edge computing and how their unique characteristic highlighted new privacy and security issues.

## 4. Security and Privacy Issues of Fog Computing

In this chapter we will go over the security issues of fog computing. We will first list possible attacks toward this technology fallowed by privacy issues that may face fog computing. Noting that countermeasures are listed later on separate chapter.

### 4.1. Attacks in Fog Computing

Fog computing technology has set new records in the modern communication world by resolving the major technical issues and complexities of cloud computing. However, this technology is also prone to numerous security and privacy threats related to data and services. Due to the varying characteristics of fog computing such as geo distribution, mobility and heterogeneity, the existing security and privacy techniques of cloud computing cannot operate in a fog computing network. Hence, new state-of-the-art security mechanisms are required to deal with the security and privacy issues of fog computing. Though, fog computing provides a number of advantages as compared to cloud computing systems, but there exist some security issues which can generate hindrance in the path of deploying modern systems using fog computing. There are many literature works proposed by different authors stating the security issues prevailing in fog computing. In some research papers, various security concerns are addressed whereas in others only a particular aspect of fog applications/architecture security is discussed.

The author of [19] briefly discussed different security issues and tried to find out the various challenging aspects related to the solutions of the fog environment with focus on data computation issues in fog environment. In research work [20], the author presented and analyzed the prevailing security and trust challenges with countermeasures proposed for those challenges. The author of [6] stated the common security gaps in fog computing network by comparing different surveys. In [21], author presented various security and privacy issues. Various intrusion detection and authentication techniques are discussed by the authors of [22]. The author of [23] gave a comprehensive overview of various trust management techniques to verify their suitability with future IoT devices.

In paper [24], author gives an overview of the prevailing security issues and challenges in fog computing. In [25], author discusses the various security and privacy issues in cloud computing, edge computing, and fog computing technologies.

The fallowing are the main security threats that affect Fog computing:

(a) Forgery: It is a type of security threat where the attacker copies someone else’s identity and behavior to deceive a security system or other people by producing fake information. Due to its fake data packets, it can also degrade the network performance by using network resources such as energy, storage and bandwidth.
(b) Tampering: In this security attack, the attackers of the network mischievously alter the data to be transmitted. It is difficult to detect this attack since the mobility of user and the wireless nature of the transmission medium may lead to failure or delay in the data transmission.
(c) Spam: Spam refers to the unwanted data that is generated by the attackers including fake data gathered from users and extra information. Spam leads to consumption of important network resources, privacy breach and misleading.
(d) Sybil: Sometimes, the network attackers use fake identity to control the effectiveness and performance of fog computing and affect the reliability of the nodes. This is called Sybil attack. The attackers create such fake crowd-sensing reports which are completely untrustworthy. Moreover, they are also able to expose the personal information of a legitimate user.
(e) Jamming: In this type of attack, the network intruders generate a huge amount of data packets to jam the transmission channels and occupy the resources for a longer time period in order to prevent the legitimate users from having access to a reliable and efficient transmission medium.
(f) Eavesdropping: In eavesdropping, the attacker listens to the confidential data of genuine users from the transmission channel without them knowing. This attack is very common if the encryption technique applied over the confidential data is not efficient.
(g) Denial of Service (DoS): This is a type of attack is a famous attack that could affect many environments including but not limited to fog computing in which fake data is sent towards the fog nodes by attackers and these nodes are flooded with innumerous fake requests so that they remain unavailable for the legitimate users. Such attacks use up resources of the network such as bandwidth, battery, time, etc. which leads to the performance degradation of fog network as they have limited resources.
(h) Man in the Middle: Man-in-the-middle is an attack where the attacker stands in between the communicating nodes to overhear and steal the useful communication of the genuine users without them knowing as they think the data is being exchanged with the legitimate receivers directly. A temporary scenario is created by the attacker in this case where he comes in between the communicating parties.
(i) Collusion: This type of security attack consists of a two or more groups that collude cooperatively to mislead and cheat the legitimate users. In order to increase the impact of the attack, these group trick and attack a group of fog nodes or IoT nodes or fog nodes with IoT nodes or IoT nodes with cloud nodes.
(j) Impersonation: In impersonation attack, as the name shows, the attacker acts like a genuine server to trick the legitimate users by offering fake or malicious services to them to make them believe that they are communicating with the real fog node or server. This way, the attacker steals all the confidential data from the legitimate user’s system without their consent.
(k) Virtual Machine Attack: Virtual machine attack is an attack where a hacker secretly takes control over the hypervisor that forms a virtual environment within a virtual machine. There are 4 different modes of attack on a virtual machine: guest to host, virtual machine to virtual machine manager, virtual machine to virtual machine, and virtual machine manager outside attacks.
(l) Side-channel Attack: This is an attack in which the cryptography of the device is unlocked by gathering information about the applied cryptographic algorithm.
(m) Session Hijacking: In session hijacking, the attacker intercepts and hijacks the user session in order to get access to the user confidential data and services.

Table 1 shows several security attacks toward Fog computing with some related contribution related to these attacks in the literature.

**Figure 4:**
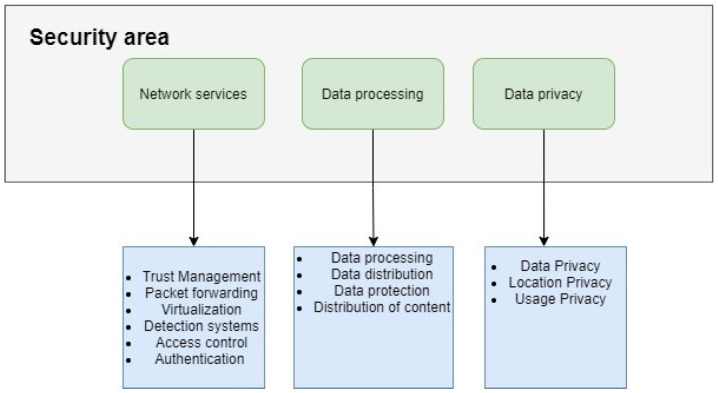
Security areas in fog computing.

Based on the type of security threat any fog computing attack land in one of three fields which are network services where the attacker aims to modify the way the network is formed or causing failure in delivery of packets in the network, Data processing attacks which affect distribution, protection and content of the data in the network and finally device privacy attacks where the attacker aim to affect the privacy of the actual device including location, usage and identity privacy. Figure 4 shows the main three areas of attacks in fog computing.

### 4.2. Privacy Issues in Fog Computing

Privacy is becoming a serious challenge in fog computing as the confidential data of users is exchanged, gathered, processed, and transmitted over fog nodes. Each user wants its data to be safe and secure over the wireless medium but, unfortunately, it is very difficult to preserve the privacy of user’s confidential data due to the presence of malicious users and intruders in the network. It is very important to maintain privacy from the perspectives of both user and the provider. As, the application processing of fog computing is carried out in the user’s device, keeping the privacy of user’s information is the first priority. According to [24,37], there are six major aspects of privacy issues:

(a) User Privacy: Fog computing network contains large number of IoT enabled devices that are inter-connected via sensors or wireless system. The job of IoT devices is to generate sensitive data and transmit it to fog nodes for processing. This sensitive data includes personal information, smart home automated data, healthcare information, business information, etc. and all of this data can be stolen by the intruder with a weak security system.
(b) Identity Privacy: The identity of a user is extremely vulnerable of getting disclosed while having authentication of fog nodes as each user has to provide their identity related information to the nodes including name, phone number, home address, passport number, license ID etc. in order to get verified.
(c) Data Privacy: The confidential data of a user can get exposed to a network attacker who is trying to steal user’s personal data from the transmission medium or relay nodes. This information consists of user’s personal address, preferences and political data. For example, the online system of voting can put the political preference of users at risk. The privacy of such data is very critical.
(d) Usage Privacy: Usage privacy refers to the pattern in which the user accesses the services of the fog computing network. This pattern can help the intruder in knowing when a user is accessing the channel for data transmission and when he is not communicating. On the basis of this pattern, the intruder launches an attack on the user’s confidential information or the channel to make it appear as ‘busy’ for the legitimate user.
(e) Location Privacy: Nowadays, each mobile application asks for access to device current or saved location along with access to user mobile’s internal data such as gallery. Due to this, the user has to sacrifice their location privacy in order to enjoy the internet services. However, little do they know that their location privacy is extremely critical information which if once obtained by the attacker can enable them to know the trajectory of the user. Hence, the location privacy of a user must be kept secure at all costs.
(f) Network Privacy: Wireless connections are always at risk due to security and privacy attacks which is a highly considerable issue. Moreover, the maintenance of fog nodes is also costly and challenging since they are present at the edge of the Internet, where network configurations are carried out manually. Hence, privacy breach is not difficult to occur. To resolve this issue, an encryption technique such as Home-Area Network can be quite useful.

In Table 2, below, we have shown some of the privacy issues of fog computing network [25] and the discussed privacy aspects:

The next chapter will discuss security and privacy issues for edge computing in details.

## 5. Security and Privacy Issues of Edge Computing

Like fog computing, edge computing technology is also facing severe security challenges due to which the confidential data of users is at stake. In [38], the authors proposed salable distributed machine learning approach to detect attacks towards edge computing environment. In [39], the author surveys the existing security threats and privacy challenges along with the cryptographic techniques and countermeasures in edge computing network.

We can summarize the main challenges of edge computing as fallow. First, normally nodes of edge are connected to a very a large number of IoT devices which have a limited resource in addition to different internal components this leads to having different types of routing protocols to disseminate messages this difference in the components may lead to some security issues. Which may lead to having several challenges in terms of access control in IoT environment. Another challenge that rise some concerns regard security in edge computing is key management of communications. While edge computing can provide end to end communication of IoT devices relying on different routing protocols; confidentiality and integrity of the data is still concerning. Therefore, a special key management and distribution mechanism may have to be designed to handle this concerns. In research work [40], the author gives a detailed analysis of security challenges in edge computing from five aspects: key management, access control, privacy protection, attack mitigation and anomaly detection. Furthermore, this paper presents the work achieved in edge computing security into five categories along with the current research situation in these categories. In Table 3, we have shown a summary of the data security and privacy challenges categorized with respect to the edge computing architecture [39].

There are different factors that lead to security and privacy issues in edge computing networks that put the user’s personal data at risk, as discussed below:

(a) In edge computing, edge nodes exist nearer to users which result in reception of large amount of sensitive data. If any of these data are stolen, it can result in an alarming consequences.
(b) Edge computing possess limited network resources, as compared to cloud computing, due to which they do not support complex encryption algorithms.
(c) Edge computing network consists of dynamic environment which is constantly changing. As a result of this, attackers can easily become part of the group. Moreover, it is very difficult to create security rules for such dynamic network.

The attacks on edge computing are as follows:

- Eavesdropping: Similar to the fog computing, the eavesdropping attack in edge computing consists of an eavesdropper who can hide itself and maliciously monitor the activity on the channel to steal or overhear the confidential data.
- Denial of Service (DoS) Attacks: Denial of service attack, just like in fog computing, allows the intruder or hacker to take control over the system or network and make its access unavailable for the legitimate user by sending a large amount of requests that jam the network.
- Distributed Denial of Service (DDoS) Attack: It is an attack in which the goal of the attacker is to interrupt the normal services provided by different servers on the basis of distributed resources like cluster of compromised edge devices. This attack happens when an attacker constantly sends innumerous packets towards the victim’s device from the compromised distributed devices as a result of which exhausts the hardware resources of the victim to handle any other packet and, therefore, fails to fulfill any genuine request in time.
- Data Tampering Attack: In data tampering attack, the attacker can alter the data transmitted over the communication channel or saved in the storage.
- Service Manipulation: It is an attack in which the adversary takes control over the edge data center, and as a result it can misrepresent or alter the services.
- False Data Injection: False data injection is an attack in which the attacker injects a false code in the network which gathers all the stored data from the database and brings it to the attacker [39].
- Physical Attack: This attack occurs when the physical protection of the edge infrastructure is weak or careless. Physical attack will affect the services in particular geographical areas as the deployment of edge servers is distributed [39].
- Rogue Gateway: Rogue gateway is an attack launched by attackers where they inject high traffic into the entire edge computing network infrastructure and the consequences are the same as the man-in-the-middle attack.

In edge computing, if the invaders have acquired sufficient control privilege over the edge data center, they can act as a genuine administrator or misrepresent the services. This enables the invaders to launch several attacks on the network such as man-in-the-middle, DoS, etc.

There are several privacy issues in edge computing include:

- Weak security techniques and algorithms for system protection which can increase the vulnerability of the network that will pave way for the malicious users to invade over the system or add external nodes to collect data in unauthorized manner.
- Unsafe communication sessions between devices.
- Difficult recovery and data backup when the system outage occurs.
- No specific pattern of update reception and implementation on the system.
- Lack of proper network visibility.
- Lack of user’s selective data collection.

Now that we have some good understanding and knowledge of the security and privacy issues of both fog and edge computing the next chapter will illustrate some countermeasures to handle such attacks and mitigate the risk caused by it.

## 6. Fog and Edge Computing Attacks Countermeasures

In this chapter we will discuss the countermeasure for the attacks that may affect fog computing and edge computing in details starting with fog computing.

### 6.1. Countermeasures for Attacks in Fog Computing

Fog computing is currently at a beginner’s stage and there is still a long way to go facing different challenges due to its unique features. It utilizes idle resources created by user devices which are not examined carefully by any standard body, as a result of which security and privacy concerns are raised in the fog network. Therefore, secure and fast authentication mechanisms are need of the time for fog as many devices are part of the fog application processing.

Some of the countermeasures proposed for mitigating the malicious attacks and privacy issues include:

- Efficient Encryption Techniques: With the help of efficient encryption techniques, the privacy issue can be resolved as the attackers will be unable to decode the complex encryption algorithms. However, the developers should consider one fact while developing an encryption technique that as technology is advancing, the attackers are also getting equipped with modern systems and techniques. Hence, they are always one step ahead of the developers as the modern technology would help them to decode any encryption algorithm.
- Decoy Technique: It is a security technique that is used to authenticate the data of a user present in the computing network. It replaces the original information with the fake one which is then provided to the attackers. When an attacker causes a security breach in the system, it finds a fake information file in place of the original file. This file is known as the decoy file and the proposed method is called as decoy technique. The decoy files are formed in the start to ensure improved security. The system hides the original data, which can only be accessed by the authenticated users, and replaces it with the decoy file by default for system intruders.
- In [41], the author proposes a data privacy preserving technique in fog computing network by using decoy technique. This technique is split into two steps where in the first stage, both verified and the unverified users will be provided decoy data file by default. Furthermore, in the second step, the verified user will be given access to the original data file in the system by passing all the security authentication challenges. When any abnormal activity is observed in the network, the system quickly generates a decoy file in the network with the help of decoy technique which is then sent towards the intruder looking same as the original file. The decoy file contains fake and bogus data. The legitimate user will identify the fake information right away, whereas the attacker will be confused with it.
- Modified Decoy Technique: It is a modified version of the original decoy technique in which the attackers are given fake data and nodes which are run by the attackers and in the meantime, information regarding identity (such as Mac address) is collected by the hidden files.
- Intrusion Detection System (IDS): Intrusion detection system (IDS) is employed in fog computing to detect and protect from attacks including DoS, insider attacks, port scanning attacks, flooding attacks on virtual machine, man-in-the-middle attacks, hypervisors and many other [42]. To secure the fog system, a perimeter IDS system is deployed that is able to coordinate different IDS in the fog system [43]. However, it can create several challenges as well for ensuring delay-less requirements. The author of [44] proposes an Intrusion Detection and Prevention (IDPS) mechanism to deal with the man-in-the-middle attacks in the fog network. This mechanism consists of IDS nodes that detect any anomaly happening in the network by monitoring it after regular time intervals. In case of a malicious node detection, that node is isolated from the network.
- Authentication Schemes: Authentication allows verification of user’s identity by verifying user’s given credentials that whether they match with information present in the database through an authentication server. This help in defending against the intrusions of malicious entities. Fog computing network enables users to access the fog services from the fog infrastructure if the user is well authenticated from the system first in order to be a part of the network processing infrastructure. In case of an unsecure authentication scheme, the entire cloud, fog nodes, and user’s device can be caused harm by network attackers, which is one of the significant security concerns.
- In research work [45], author proposes a secure mutual authentication scheme for fog computing, that enables authentication of any fog user with the fog nodes mutually in a fog network. The author of [46] proposes a multi-tier authentication technique that allows secure Login in fog computing.
- Blockchain Security for Fog Computing: The concept of blockchain was introduced for secure cryptocurrency application of Bitcoin. However, with the passage of time, the researchers realized that blockchain can also be used to secure cloud and fog computing networks with its extraordinary security features. Therefore, the security of fog environment can be enhanced by employing blockchain technique. Given below are some useful blockchain feature with context of fog computing [41]:
  − Reduces failure of single point.
  − Allows network transaction with highly secure encryption algorithm.
  − Capable of tracking node status efficiently.
  − Immutable technology.

Blockchain technology can prevent various malicious attack in fog network including man-in-the-middle attack, DoS attack and data tampering [47,48].

In Table 4, we have summarized the countermeasure used for securing and preserving the privacy of fog computing environment.

### 6.2. Countermeasures for Attacks in Edge Computing

Since Edge computing as illustrated in this paper have special features different security solutions preformed on cloud computing service cannot be effective with edge computing some of proposed solutions that lands with the unique characteristics of edge computing are:

- Edge Node Security: Same level of security must be applied on all nodes of the edge network to ensure proper safety protocols. In case of different security levels, the attacker may break through the node having weak security algorithm causing system’s degradation. Moreover, different security levels can also cause trouble for the system operators in determining which node has weak security barrier that allowed security breach.
- Full-time Monitoring: In order to secure a network from malicious users, it is necessary to constantly keep an eye on all the edge nodes and provide network visibility to the users in an interactive interface.
- Proper Encryption: With the advance in modern technology, new state-of-the-art encryption algorithms are being proposed that are very complex to decode. These algorithms consist of a secret key which is properly secured and shared between the legitimate sender and receiver. This secret key allows the genuine users to decrypt the algorithm and access the data.
- Intrusion Detection System: It is a system which informs the user about any anomaly or unauthorized access it detects in the system.
- User Behavior Profiling: User behavior profiling refers to the observation, monitoring and maintenance of general behavior of the users so that any activity different from the normal behavior will help in determining the presence of a malicious user. Hence, the users will be informed about the abnormal activity.
- Cryptographic Techniques: Cryptographic techniques are being proposed to deal with the security attacks launched by hackers and intruders. These techniques mostly consists of a secret key that is only shared between the sender and the receiver. This secret key is used to decrypt the received message. If an intruder succeeded in acquiring this secret key from the transmitted packets over the communication channel then he would be able to steal the data in the message.
- Data Confidentiality: To deal with the different privacy issues that are caused by the illegal data operations, loss of data, data manipulation, data breach etc. by network attackers, various data confidentiality mechanism are proposed based on encryption algorithms. In paper [54], the author proposes a privacy-preserving mechanism named as QueryGuard that is a latency-aware query optimization technique. This technique achieves two fold objective: first is that it tackles the privacy-aware distributed query processing issue and second is that it optimizes the queries for delay-free communication. It achieves better performance results in terms of computation time, memory usage as compared to conventional query optimization schemes.

In survey [55], the author gives a comprehensive overview of the most basic attacks in edge computing including DoS, DDoS, side channel attack, malware injection attacks, etc. along with the defense mechanisms that can be applied in edge computing network. The author of [56] formulated a detection scheme for side-channel attacks that can detect the abnormal cache activity on edge servers. Numerous other defense mechanisms are proposed for edge networks but, unfortunately, the existing security and privacy-preserving mechanisms are unable to be employed in edge computing network due to its mobility, large number of edge nodes, and dynamic environment.

These mechanism run well for cloud computing but provide inefficient results in case of edge computing. Privacy for edge nodes needs adaptive frameworks that can dynamically choose the most suitable privacy scheme based on the communication environment and edge nodes involved [57]. Hence, this area of communication still needs a large amount of research and consideration. Table 5 shows main security attacks and some possible privacy-preserving countermeasures in edge computing.

## 7. Future of Fog and Edge Computing and How That Will Affect IoT and Smart Cities

Fog and edge computing technologies are going to revolutionize the current picture of the wireless communication networks, with their efficiency, effectiveness, delay-free services, real-time applications and performance enhancement capabilities. Not only this, but these technologies are also expected to increase the performance efficiency and service delivery of other technologies such as IoT.

IoT technology has received immense attention not only from the academic side but from the industrial side as well. It is basically the upcoming new era of ‘connectivity everywhere’. According to the author of [58], it is estimated that more than 20 billion devices would be connected by the end of 2020 that will be present in various business organization, industrial departments and consumer side. As per the article [59], there will be more than 21 billion devices connected by the year 2025. As the technology is advancing, the IoT is flourishing at a great pace with increased number of sensors that are assigned to various devices to efficiently handle the large amount of data being generated and store it on regular basis.

The applications of IoT require cloud technology for processing. However, as the number of IoT devices is increasing, so is the data being generated by them due to which the IoT devices can no longer depend on any central entity such as cloud computing to perform processing of such large amount of data, rather it requires a technology that can not only handle its large amount of data and service applications but also manage and control numerous sensors, actuators, device users, operations and connectivity by bringing processing facilities nearer to users. The traditional cloud computing is unable to solve the issues related to time sensitivity and connectivity. Whereas in IoT, there are large number of areas where even a delay of a microsecond can cause huge consequences like in the field of telemedicine, healthcare centers, vehicle-to-vehicle communication, security departments and many more. Hence, fog computing is being considered to be a better choice for employing with IoT technology.

The devices present on the edge generate data from their allotted areas and transmit to nearest fog nodes for analysis and decisions. The advantage of using fog computing is that it can manage IoT devices along with solving the processing limitations of time-sensitive applications in cloud computing. Fog nodes exist at the edge of the network to serve edge users with high quality services and zero delay. This allows real-time processing, storage and networking facilities to be provided to the users at edge level. IoT offers a number of suitable solutions for application such as waste management, smart traffic signal system, logistic control system, emergency services and industrial area. The two most attractive fields of IoT are smart healthcare devices and wearable sensors. Due to the ability of edge processing of fog computing technology, it has various functionalities in smart application such as smart healthcare system, smart transportation system, smart city, smart homes, smart vehicle, augmented and virtual reality and many other smart real-time applications. Fog computing technology is highly being employed in healthcare departments because the data generated by healthcare applications is time-sensitive which requires quick processing. The author of [60] proposed a smart healthcare system based on fog to control the occurrence of Chikungunya virus. In this system, the wearable IoT sensor collected health data and medical data of the patient. According to [61], many challenges of IoT can be resolved with fog computing technology, as shown in Table 6.

## 8. Conclusions

In this paper, we discussed and surveyed the security and privacy aspects of two new paradigms of cloud computing which are fog computing and edge computing. Unlike cloud computing, fog and edge computing have special characteristic that lead to having some new security and privacy concerns. We showed different type of attacks that are shared between the two technologies such as shared resources and DDoS attacks as well as some of the unique attacks for each environment. We also provided in this paper some possible countermeasures that could mitigate some of the listed attacks. Through researching the vulnerability of these technologies we found that the security aspect of the two technologies are far from satisfied yet and there is a lot of potential research areas to be covered. In Table 7, we conclude a list of the main threats for the two technologies in addition to referring some work in the literature that discussed these threats and the attacks related to these threats. In [62], the authors suggested a pattern for fog computing that in our opinion could be enhanced and add security patterns to it to create a security reference architecture for fog computing.

## Figures and Tables

**Table 1:** Security attack in fog computing with some researches contribution.

Security Attack	Research Contribution
Forgery	In [26], the authors suggested privacy preserving authentication scheme that provide access control which address the forgery attack.
Tampering	In [27], the authors suggested reliable trust computing mechanism (RTCM) based on fog computing fusion. Their suggested solution aims to provide high level of integrity for the data in fog environment.
Sybil	In [28], the authors suggested a Sybil attack detection mechanism for the cloud computing environment that also could be used with fog computing.
Jamming	In [29], the authors discussed jamming issues in fog computing.
Eavesdropping	In [30], the author suggested some techniques that can detect and prevent this type of attacks.
Denial of Service	In [31], the authors provided a comprehensive overview of this type of attacks and how they could affect fog computing. In [32], the author suggested lightweight mechanism to mitigate this type of attacks.
Man-in-the-Middle	In [33], the author discussed this type of attack in fog computing and how it could be detected.
Collusion	In [34], the authors identified different privacy framework to prevent this type of attacks.
Impersonation	In [32], the authors discussed this type of attacks and provided a technique to prevent it.
Side Channel Attack	In [35], the author suggested a system with resistant to side channel attacks in fog computing.
Session Hijacking	In [36], the author discussed a way to prevent this type of attacks in fog and edge environment.

**Table 2:** Privacy and issues in fog.

Sr. No.	Privacy Aspects	Privacy Issues
1.	User Privacy	Limited Network Visibility
2.	Identity Privacy	Inefficient Attack Detection techniques
3.	Data Privacy	Unavailability of user selective data collection
4.	Usage Privacy	Issues of Virtualization
5.	Location Privacy	Issues of Multitenancy
6.	Network Privacy	Suspected fog nodes

**Table 3:** Security Attack in Edge Computing Architecture.

	Core Side	Edge Servers	Edge Network	Mobile Edge Devices
Challenges	Privacy Leakage	Privacy Leakage	DoS	Data Injection
	Data Tampering	DoS	Man-in-the-middle	Service Manipulation
	DoS	Privilege Escalation	Rogue Gateway	
	Service Manipulation	Service Manipulation		
		Rogue Data Center		
		Physical Damage		

**Table 4:** Security attacks and privacy-preserving countermeasures.

Countermeasures	Brief Description
Efficient Encryption Techniques	With efficient encryption techniques, the privacy issue can be resolved as the attackers will be unable to decode the complex encryption algorithms. the authors of [49] discussed the topic of encryption and how to apply it to fog environment.
Decoy Technique	It is a security technique that is used to authenticate the data of a user present in the computing network by replacing the original information with the fake one which is then provided to the attackers. The authors of [50] suggested a method that deal with decoy techniques with the help of user profiling.
Intrusion Detection System	It is employed in fog computing to detect and protect from attacks including DoS, insider attacks, port scanning attacks, flooding attacks on virtual machine, man-in-the-middle attacks, hypervisors etc.the authors of [51] proposed a lightweight intrusion detection system based on a vector space representation using a Multilayer Perceptron (MLP) model.
Authentication Schemes	It allows verification of user’s identity by verifying user’s given credentials that whether or not they match with information present in the database through an authentication server. In [52], the authors proposed a secure identity based anonymous authentication scheme for mobile edge computing.
Blockchain Security	Blockchain allows network transaction with highly secure encryption algorithm and reduces failure of single point. In [53], the authors discuessed integration of blockchain with edge computing and the challenges facing such integration.

**Table 5:** Security attacks and privacy-preserving countermeasures in edge.

Countermeasures	Brief Description
Edge Node Security	Same level of security must be applied on all nodes of the edge network to ensure proper safety protocols. In case of different security levels, the attacker may break through the node having weak security algorithm.
Full-time Monitoring	It refers to constantly keep an eye on all the edge nodes and provide network visibility to the users in an interactive interface.
Proper Encryption	It involves a complex algorithm or a secret key which is properly secured and shared between the legitimate sender and receiver that allows the genuine users to decrypt the algorithm and access the data.
Intrusion Detection System	It is a system which informs the user about any anomaly or unauthorized access it detects in the system.
User Behavior Profiling	It refers to the observation, monitoring and maintenance of general behavior of users so that any activity apart from the normal behavior will determine the presence of an attacker.
Cryptographic Techniques	These techniques are used to deal with the security attacks launched by hackers and intruders by using a secret key.
Data Confidentiality	This mechanisms deal with different privacy issues that are caused by the illegal data operations, loss of data, data manipulation, data breach etc. by network attackers.

**Table 6:** Summary of IoT challenges and fog solutions.

Challenges in IoT	Solution Offered by Fog
Security Challenge	Fog network is able to scan malware and determine the security status of surrounding IoT devices. It is also able to act as proxy to update software and detect threats timely.
Delay Constraints	Fog can perform various time-sensitive computation tasks.
Network Bandwidth Constraints	Fog enables hierarchical data processing for transferring data from cloud to IoT devices.
Uninterrupted Services	Fog promises uninterrupted services even if there is some connection issue.
Resource Constrained Devices	Fog is able to reduce device complexity, cost and consumption of power when certain operations cannot be delivered to the cloud.

**Table 7:** Main threats of fog and edge computing and related researches.

Threat	Related Description	Related Resources
Access control	This includes all attacks that give the attacker access to sensitive or private data in unauthorized manner.	In [63], the authors suggested a solution that could be applied to Fog computing where data get encrypted at the fog end using attribute-based encryption and multiple policies. In [64], the authors suggested a solution that could be applied to Fog computing where data get encrypted at the fog end using attribute-based encryption and multiple policies. In [65], the authors proposed a policy based management of resources in fog computing to support secure collaboration between different users without interference. In [45], the authors proposed a secure authentication method that could be applied to fog and edge environment which allow authenticating users mutually in the network.
Denial of service (DOS)	In this type of attacks fake data is sent towards the fog nodes by attackers and these nodes are flooded with innumerous fake requests so that they remain unavailable for the legitimate users.	In [31], the authors suggested reinforcement learning methods to mitigate Denial of service attacks towards edge servers. In [66], the authors discussed using blockchain as a service in order to defend Denial of service this solution could be applied to Fog computing.
Virtualization threats	This type of attacks includes all attacks related to virtual machine including shared resources attacks where attackers try to take over some of the resources in unauthorized manner as well as migration attacks where attacker try to compromise the virtual machine during migration process.	In [67], the authors suggested using identify mechanisms and intrusion detection system for edge computing in order to countermeasures attacks aimed toward virtual machines in edge computing such as creating fake edge service. In [68], the authors suggested a secure migration mechanism for virtual machine. In [69], the authors discussed several issues that face virtualization the hardware that could apply to fog and edge computing.
Trust Management	This includes all attacks where attackers gain a trust allowing them to communicate with different nodes and establish unauthorized connection to the network. Some examples of such attacks are: Self-promotion attack, Bad-mounting attack and on-off attack.	In [22], the authors discussed Rogue Fog devices and how they could create a threat toward the environment. In [63], The authors proposed a reputation system model that could be applied to both edge and fog environment through applying distributed pooling algorithm to check validity of the model and communications. In [70], the authors proposed a lightweight trust evaluation mechanism to employ fusion of multi sourced feedback in order to evaluate trust level in the environment.
